# Macrophage migration inhibitory factor facilitates production of CCL5 in astrocytes following rat spinal cord injury

**DOI:** 10.1186/s12974-018-1297-z

**Published:** 2018-09-04

**Authors:** Yue Zhou, Wei Guo, Zhenjie Zhu, Yuming Hu, Yingjie Wang, Xuejie Zhang, Wenjuan Wang, Nan Du, Tiancheng Song, Kaini Yang, Zongyu Guan, Yongjun Wang, Aisong Guo

**Affiliations:** 1 Department of Rehabilitation Medicine, Affiliated Hospital of Nantong University, Nantong, 226001 People’s Republic of China; 2 Key Laboratory of Neuroregeneration of Jiangsu and Ministry of Education, Co-innovation Center of Neuroregeneration, Nantong University, Nantong, 226001 People’s Republic of China; 3 School of Basic Medical Sciences, Nanjing Medical University, Nanjing, People’s Republic of China; 4 Medical College, Nantong University, Nantong, 226001 People’s Republic of China

**Keywords:** MIF, CCL5, Spinal cord, Astrocyte, Inflammation, Chemokines

## Abstract

**Background:**

Astrocytes act as immune effector cells with the ability to produce a wide array of chemokines and cytokines in response to various stimuli. Macrophage migration inhibitory factor (MIF) is inducibly expressed in injured spinal cord contributing to excessive inflammation that affects motor functional recovery. Unknown is whether MIF can facilitate inflammatory responses through stimulating release of chemokines from astrocytes following spinal cord injury.

**Methods:**

Following the establishment of the contusion spinal cord injury rat model, the correlation of chemokine (C-C motif) ligand 5 (CCL5) expression with that of MIF was assayed by Western blot, ELISA, and immunohistochemistry. Immunoprecipitation was used to detect MIF interaction with membrane CD74 receptor. Intracellular signal transduction of MIF/CD74 axis was analyzed by transcriptome sequencing of primary astrocytes and further validated by treatment of various inhibitors. The effects of CCL5 released by astrocytes on macrophage migration were performed by transwell migration assay. The post-injury locomotor functions were assessed using the Basso, Beattie, and Bresnahan (BBB) locomotor scale.

**Results:**

The protein levels of chemokine CCL5/RANTES were remarkably increased in the astrocytes of rat injured spinal cord, in parallel with the expression of MIF. Treatment of MIF inhibitor 4-IPP in the lesion sites resulted in a significant decrease of CCL5 protein levels. In vitro study revealed MIF was capable of facilitating CCL5 production of astrocytes through interaction with CD74 membrane receptor, and knockdown of this receptor attenuated such effects. Production of CCL5 in astrocytes was significantly blocked by inhibitor of c-Jun N-terminal kinase, rather than by those of ERK and P38. Recombinant CCL5 protein was found to be more effective in promoting migration of M2- compared to M1-type macrophages.

**Conclusion:**

Collectively, these data reveal a novel function of MIF in regulation of CCL5 release from astrocytes, which in turn favors for recruitment of inflammatory cells to the injured site of the spinal cord, in association with activation of excessive inflammation.

**Electronic supplementary material:**

The online version of this article (10.1186/s12974-018-1297-z) contains supplementary material, which is available to authorized users.

## Background

The central nervous system (CNS) can activate innate immune responses by sensing pathogens or tissue damages [1]. Upon acute traumatic spinal cord injury, the resident immune and glial cells are rapidly motivated to release various cytokines and chemokines, which further induce leukocyte infiltration to the injury sites [2, 3]. Astrocytes are the most abundant glial cell contributing to the functional homeostasis of the healthy CNS. They can also receive and integrate information from synapses, other glial cells, and the blood vessels and as a consequence generate complex outputs that control the neural circuitry and coordinate it with the local microcirculation [4]. Astrocytes have been shown to participate in local inflammatory responses [5–8]. In the context of inflammatory or otherwise pathological conditions, the activated astrocytes proliferate and produce a wide variety of cytokines and chemokines, including CCL2 (monocyte chemotactic protein-1), CCL5 (RANTES), and CXCL8 (IL-8), which are associated with the recruitment of immune cells of alternative activation status [5, 9]. These recruited microglia/macrophages play either a pro-inflammatory (M1) or anti-inflammatory (M2) role as seen in a spinal cord injury model or neuroinflammatory diseases like multiple sclerosis (MS) [10]. Chemokine (C-C motif) ligand 5 (CCL5) may serve to amplify inflammatory responses by facilitating the recruitment of inflammatory cells into autoimmune lesions [6, 11, 12]. As such, astrocyte-released cytokines and chemokines which contribute to inflammatory responses will inevitably exacerbate the neuropathological changes following CNS injury.

Macrophage migration inhibitory factor (MIF) is a pro-inflammatory cytokine produced by a variety of cells and tissues, including monocytes, the anterior pituitary gland, T-lymphocytes, hepatocytes, and keratinocytes [13, 14]. MIF has been found to be constitutively or inducibly expressed in astrocytes, neurons, microglia, ependymal cells, and epithelial cells of the choroid plexus in the CNS [14–17]. Besides its roles of inducing neuronal death and activating inflammatory episodes in microglia, MIF is capable of promoting proliferation and inflammatory cytokine production of astrocytes [16, 18]. Deletion of MIF has been shown to promote functional recovery after compression-induced spinal cord injury [17], suggesting that MIF plays key roles in the neuropathogenesis of injured spinal cord. Astrocytes are the main sources of chemokines involved in recruitment of inflammatory cells; whether MIF is able to induce the release of chemokines from the cells remains unclear.

Astrocytes are endowed with the ability to secrete cytokines through expressing an array of receptors involved in innate immunity, such as Toll-like receptors and nucleotide-binding oligomerization domains [19, 20]. Also, the cells express CD74 surface receptor which can interact with MIF to elicit inflammatory responses after spinal cord injury (SCI) [18]. As MIF/CD74 axis has been found to activate intracellular MAPKs of astrocytes, an intermediate signaling essential for regulation of CCL5 and other chemokines [21, 22], MIF/CD74 axis is therefore postulated to mediate the production of CCL5 in astrocytes, which has been identified to promote inflammatory cell recruitment. In the present study, we analyzed the correlations between expression of MIF and CCL5 in the injured spinal cord of rat. We further investigated MIF-induced production of CCL5 in the astrocytes, as well as the underlying molecular mechanism. Our results have revealed that MIF affects pathological changes of the injured spinal cord through not only activation of astrocyte inflammatory responses, but also promoting release of chemokines.

## Methods

### Animals

Adult male Sprague–Dawley (SD) rats, weighing 180–220 g, were provided by the Center of Experimental Animals, Nantong University. All animal care, breeding, and testing procedures were approved according to the Animal Care and Use Committee of Nantong University and the Jiangsu Province Animal Care Ethics Committee. All animals were housed in individual cages in a temperature and light/dark cycle controlled environment with free access to food and water.

### Establishment of the contusion SCI rat model

The number of animals subjected to surgical treatment was calculated by six per experimental group in triplicate. The contusion SCI rat model was prepared as the previous description [23]. Briefly, rats were anesthetized with an intraperitoneal injection of 10% chloral hydrate (3 mg/kg). The fur was shaved from the surgical site and the skin was disinfected with chlorhexidine. A 15-mm midline skin incision was made to expose the vertebral column. After the spinal thoracic region was exposed by separation of the dorsal muscles to the side, the spinous processes of T8–T10 vertebrae were exposed. A laminectomy was performed at vertebral level T9, exposing the dorsal cord surface with the dura remaining intact. The exposed spinal cord segment (about 3 mm in length) received a 150-kilodyne spinal contusion injury using the IH-0400 Impactor (Precision Systems and Instrumentation) injury device. The impact rod was removed immediately, and the wound was irrigated. Muscles and incisions were sutured using silk threads. Postoperative care included butorphanol administration twice a day for a 5-day period, as well as vitamins, saline, and enrofloxacin twice a day for a 7-day period. Manual expression of the bladders was performed twice a day until animals recovered spontaneous voiding.

### Cell culture

Astrocytes were prepared from the spinal cord of newborn Sprague–Dawley rats, 1–2 days after birth, and the astrocytes were isolated and cultured according to previously described methods [24]. Briefly, the cells were enzymatically dissociated using 0.25% trypsin (Gibco-BRL) for 6 min at 37 °C, and the suspension was then centrifuged at 1200 rpm for 5 min and cultured in 1:1 Dulbecco’s modified Eagle’s medium to Ham’s F-12 medium supplemented with 10% fetal bovine serum (FBS), 0.224% NaHCO_3_, and 1% penicillin/streptomycin in the presence of 5% CO_2_. A monolayer of astrocytes was obtained 12–14 days after the plating. Non-astrocytes were detached from the flasks by shaking and were removed by changing the medium. Third or fourth passage cells were rendered quiescent through incubation in the medium containing 0.5% FBS for 4 days prior to the experiments. Astrocyte phenotype was confirmed by cells exhibiting a characteristic morphology and positive staining for the astrocytic marker glial fibrillary acid protein (GFAP).

### Western blot

Protein was extracted from cells with a buffer containing 1% SDS, 100 mM Tris-HCl, 1 mM PMSF, and 0.1 mM β-mercaptoethanol, following treatment with 0.5 μg/ml rat recombinant MIF (ProSpec) for 15 min, 30 min, and 60 min, respectively. Alternatively, protein was extracted from 1-cm spinal segments of the injured site at 0 day, 1 day, 4 days, and 1 week following contusion (*n* = 8 in each time point). Protein concentration of each specimen was detected by the Bradford method to maintain the same loads. Protein extracts were heat-denatured at 95 °C for 5 min, electrophoretically separated on 10% SDS-PAGE, and transferred to PVDF membranes. The membranes were subjected to the reaction with a 1:1000 dilution of primary antibodies in TBS buffer at 4 °C overnight, followed by a reaction with the secondary antibody conjugated with goat anti-rabbit or goat anti-mouse HRP dilution 1:1000 (Santa Cruz) at room temperature for 2 h. After the membrane was washed, the HRP activity was detected using an ECL kit. The image was scanned with a GS800 Densitometer Scanner (Bio-Rad), and the data were analyzed using PDQuest 7.2.0 software (Bio-Rad). β-actin (1:5000) was used as an internal control. The antibodies used in Western blot are as follows: MIF (Abcam); p65NFκB (Cell Signaling Technology, CST), p-ERK1/2, and ERK1/2 (CST); and CD74 (Biorbyt) and β-actin (Proteintech).

### ELISA

Primary astrocytes were treated with 0–2.5 μg/ml rat recombinant MIF for 24 h, or tissue samples of spinal segments were prepared as mentioned (*n* = 6 in each time point). Cell supernatants were harvested, and cells were lysed in the buffer containing 1% SDS, 100 mM Tris-HCl, 1 mM PMSF, and 0.1 mM β-mercaptoethanol. The lysates were centrifuged at 12,000*g* for 15 min. Levels of CCL5 were assessed using the appropriate ELISA kits (BD Biosciences, R&D Systems) according to the manufacturer’s directions. Plates were read using a 96-well plate reader (Biotek Synergy2) at a 450-nm wavelength.

### Tissue immunohistochemistry

The vertebra segments were harvested from six experimental models of each time point, post-fixed, and sectioned. Sections were allowed to incubate with monoclonal MIF antibody (1:200 dilution), rabbit anti-IBA-1 antibody (1:400 dilution, Wako), polyclonal rabbit anti-CCL5 antibody (1:200 dilution, novusbio), or monoclonal mouse anti-human GFAP antibody (1:400 dilution, Sigma) at 4 °C for 36 h. The sections were further reacted with the FITC-labeled secondary antibody goat anti-mouse IgG (1:400 dilution, Gibco) or the TRITC-labeled secondary antibody donkey anti-rabbit IgG (1:400 dilution, Gibco) at 4 °C overnight, followed by observation under a confocal laser scanning microscope (Leica, Heidelberg, Germany).

### Immunoprecipitation

The primary astrocytes were washed twice with cold phosphate-buffered saline and then extracted with lysis buffer (20 mM Tris-HCl, pH 7.5, 150 mM NaCl, 1 mM EDTA, 1 mM EGTA, 1% Triton X-100, 2.5 mM sodium pyrophosphate, 1 mM β-glycerolphosphate, 1 mM Na_3_VO_4_, 1 mM phenylmethylsulfonyl fluoride, and Roche Applied Science’s complete protease inhibitors). Whole-cell extracts were centrifuged at 14,000 rpm for 20 min to remove the debris. The proteins in the supernatant were measured using a Protein Assay Kit II (Bio-Rad). For immunoprecipitation analysis, 500 μg of total cell lysates was precleared with protein A plus G-Sepharose before incubation with specific antibodies, followed by addition of protein A plus G-Sepharose. The precipitated proteins were resolved in 2× SDS-PAGE sample buffer and separated by electrophoresis on 10–12% SDS-PAGE. After being transferred onto a polyvinylidene difluoride membrane (Millipore Corp.), they were incubated with anti-MIF or anti-CD74 antibody and further with horseradish peroxidase-conjugated secondary antibody (Santa Cruz).

### Transwell migration assay

Migration of RAW264.7 cells were measured using 6.5-mm transwell chambers with 8-μm pores (Costar, Cambridge, MA) as described previously [25]. A total of 100 μl of RAW264.7 cells (2 × 10^5^ cells/ml) was transferred into the top chamber of the transwells and allowed to migrate at 37 °C in 5% CO_2_. Meanwhile, 600 μl of astrocytes (1 × 10^5^ cells/ml) was seeded into the lower chambers. After migration for 48 h, the upper surface of each membrane was cleaned with a cotton swab. Cells attached to the bottom surface of each membrane were stained with 0.1% crystal violet, imaged, and counted using a DMR inverted microscope (Leica Microsystems, Bensheim, Germany). Assays were performed in triplicate for three times. To determine the effect of CCL5 on astrocyte-stimulated RAW264.7 migration, astrocytes were stimulated with 0.5 μg/ml recombinant MIF following CCL5 siRNA interference for 24 h prior to a transwell assay. For M2 macrophage transition, RAW264.7 cells were treated with or without 20 ng/ml rat recombinant IL-13 for 2 days.

### Sequencing of mRNA

Total RNA of astrocytes following treatment with CD74-siRNA [18] or scramble for 48 h, and then with 2.0 μg/ml recombinant MIF for 12 h and 24 h, respectively, was extracted using the mirVana miRNA Isolation Kit (Ambion, Austin, TX) according to the manufacturer’s instructions. They were then selected by RNA Purification Beads (Illumina, San Diego, CA) and undergone library construction and RNA-seq analysis. The library was constructed by using the Illumina TruSeq RNA sample Prep Kit v2 and sequenced by the Illumina HiSeq-2000 for 50 cycles. High-quality reads that passed the Illumina quality filters were kept for the sequence analysis.

### Bioinformatics analysis

Differentially expressed mRNA was designated in a criterion of greater than twofold or less than twofold change in comparison with control. Function of genes was annotated by Blastx against the NCBI database or the AGRIS database (https://agris-knowledgebase.org/) with *E* value threshold of 10^−5^. Gene ontology (GO) classification was obtained by WEGO (http://wego.genomics.org.cn/) via GO id annotated by Perl and R program. Kyoto Encyclopedia of Genes and Genomes (KEGG) pathways were assigned to the sequences using KEGG Automatic Annotation Server (KAAS) online. For all heatmaps, genes were clustered by Jensen-Shannon divergence.

A reconstructed gene network was created using the Ingenuity Pathway Analysis (IPA) Software on the basis of differentially expressed genes (fold change < 0.5 for downregulated genes at 12 h and 24 h following CD74 knockdown) to investigate their regulatory pathways and cellular functions [26].

### Behavioral tests

The hindlimb locomotor function recovery was evaluated using the Basso, Beattie, and Bresnahan (BBB) locomotor scale as previously described [27], after MIF or CCL5 treatment on 0, 7, 14, and 21 days after surgery. Three well-trained investigators who were blind to the study observed the behavior of rats for 5 min. The BBB score ranged from 0 to 21 according to the rating scale. Every rat had a BBB score of 21 before surgery. The BBB score would become 0 to 1 after a successful SCI.

### Statistical analysis

Statistical significance of differences between groups was analyzed by one-way analysis of variance (ANOVA) followed by Bonferroni’s post hoc comparisons tests with SPSS 15.0 (SPSS, Chicago, IL, USA). Normality and homoscedasticity of the data were verified before any statistical analysis using Levene’s test. Statistical significance was set at *P* < 0.05.

## Results

### MIF facilitated expression of chemokine CCL5 in the astrocytes of injured rat spinal cord

**Fig. 1:**
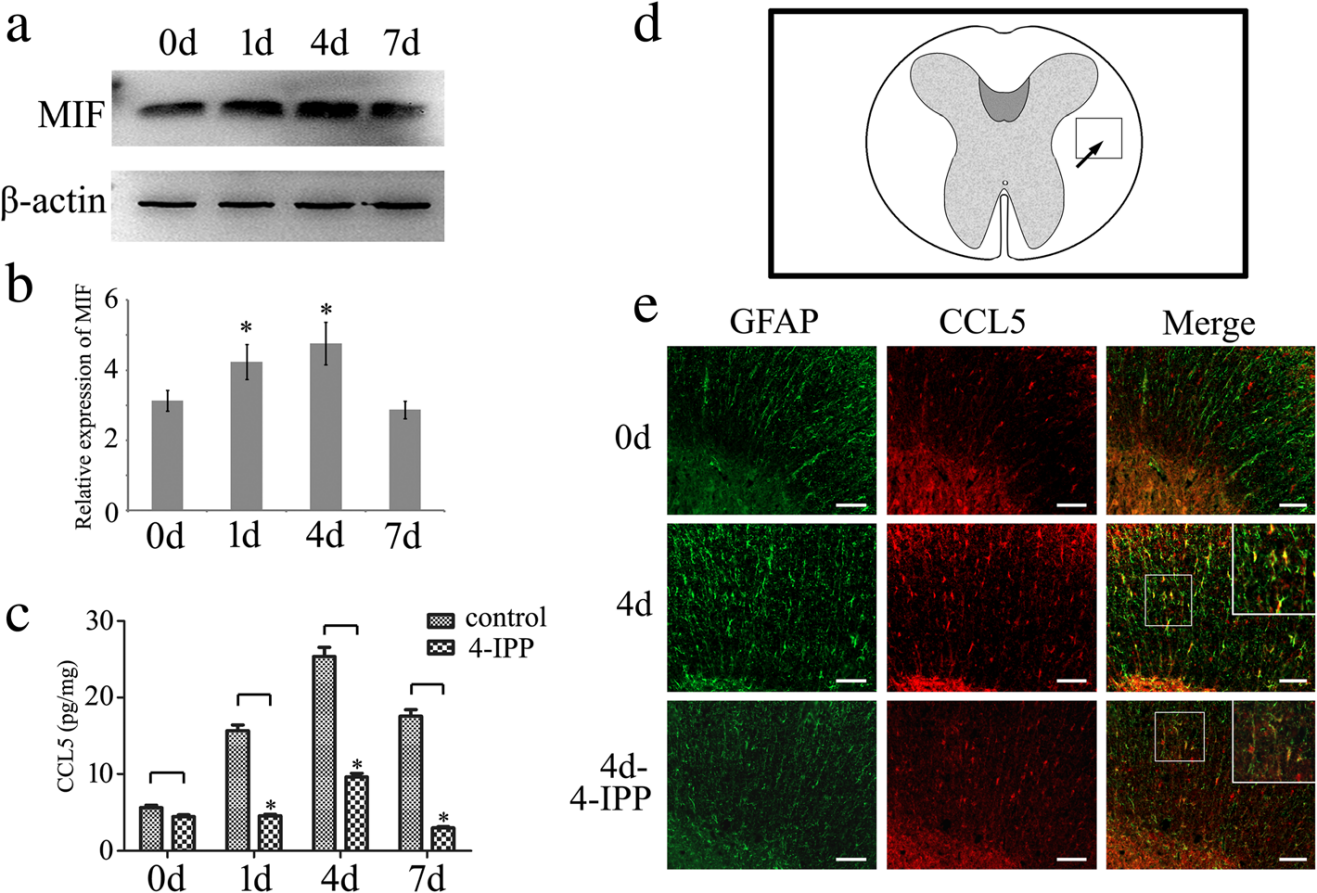
Determination of MIF and CCL5 expression following spinal cord injury. **a** Western blot analysis of MIF expression following spinal cord contusion at 0d, 1d, 4d, and 7d, respectively. **b** Quantification data as shown in **a**; quantities were normalized to endogenous β-actin. **c** CCL5 protein levels were assayed by ELISA for the injured cord at different time points with or without injection of 8 μl 4-IPP (100 mM) at the lesion sites. **d** Illustration of section sites showing immunostaining. **e** Immunostaining of CCL5 in the cross sections of rat contused spinal cord showed colocalization with GFAP-positive cells at 0d and 4d, with or without injection of 4-IPP, respectively. The rectangle indicates region magnified. Error bars represent the standard deviation (*P* < 0.05). Scale bars, 100 μm

To uncover the potential function of MIF in CCL5 expression, we firstly determined the protein levels of MIF and CCL5 in the contused spinal cord at different time points. Results revealed that CCL5 protein levels, assayed by ELISA, were synchronously increased with elevation of MIF assayed by Western blot (Fig. 1a–c). While treatment of 8 μl of 100 mM MIF inhibitor 4-IPP in the lesion site resulted in the remarkable reduction of CCL5 production (Fig. 1c). The data indicate that MIF efficiently regulates CCL5 production following SCI.

Next, we sought to understand whether MIF-induced CCL5 expression was astrocyte-related. Immunostaining showed that CCL5 was exclusively colocalized with GFAP-positive, rather than with NeuN-, IBA-1-, or Olig2-positive cells (Additional file 1). CCL5 production was significantly increased in the GFAP-positive cells at 4 days following SCI (Fig. 1d, e). Whereas injection of 8 μl 4-IPP (100 mM) at the lesion site markedly decreased the expression of CCL5 in astrocytes (Fig. 1e). The results indicate that MIF is involved in regulation of CCL5 production in astrocytes after SCI.

### Expression of CCL5 was regulated by MIF/CD74 axis in astrocytes

**Fig. 2:**
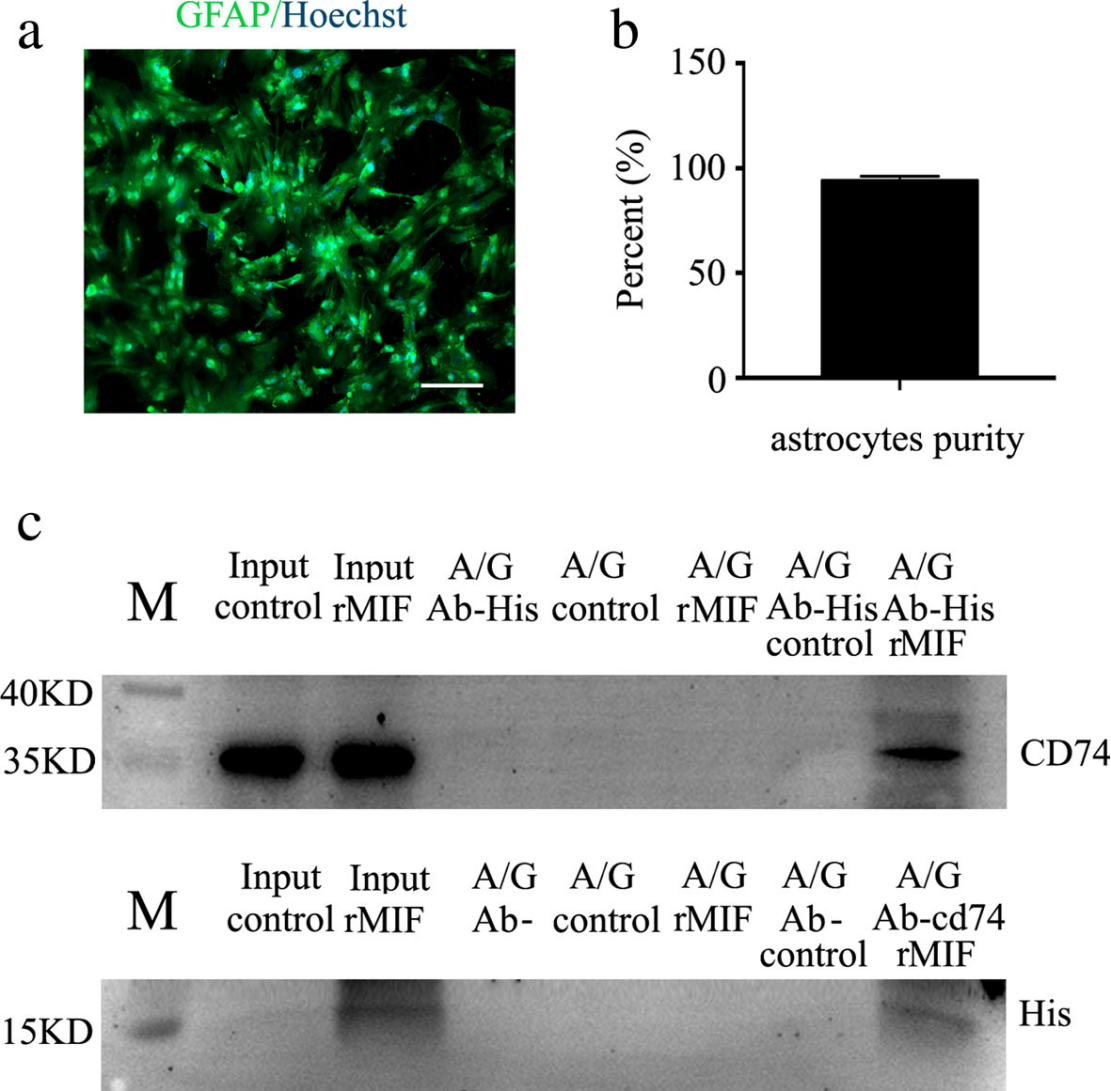
Binding assay of MIF with CD74 receptor in the primary astrocytes. **a** Purified primary astrocytes stained with GFAP and Hoechst 33342. **b** Statistical analysis of primary astrocyte purity. **c** Immunoprecipitation using anti-His or anti-CD74 antibody and detection of the components of the rMIF- or CD74-associated complexes with anti-CD74 or anti-His antibody. M marker, A/G protein A/G magnetic beads. Scale bars, 50 μm

MIF has been shown to interact with CD74 surface receptor, which forms a receptor complex with CXCR2 and CXCR4 to elicit intracellular signaling [28, 29]. To elucidate whether MIF/CD74 couple also exists in astrocytes, the rat astrocytes were firstly isolated and cultured with purity more than 95% (Fig. 2a, b). Co-immunoprecipitation was then carried out using anti-His or anti-CD74 antibody. As shown in Fig. 2c, recombinant His-tagged MIF was present in the CD74-associated complexes immunoprecipitated with anti-CD74 antibody, so was CD74 in the His-associated complexes. The results indicate that MIF can bind with CD74 surface receptor of astrocytes.

**Fig. 3:**
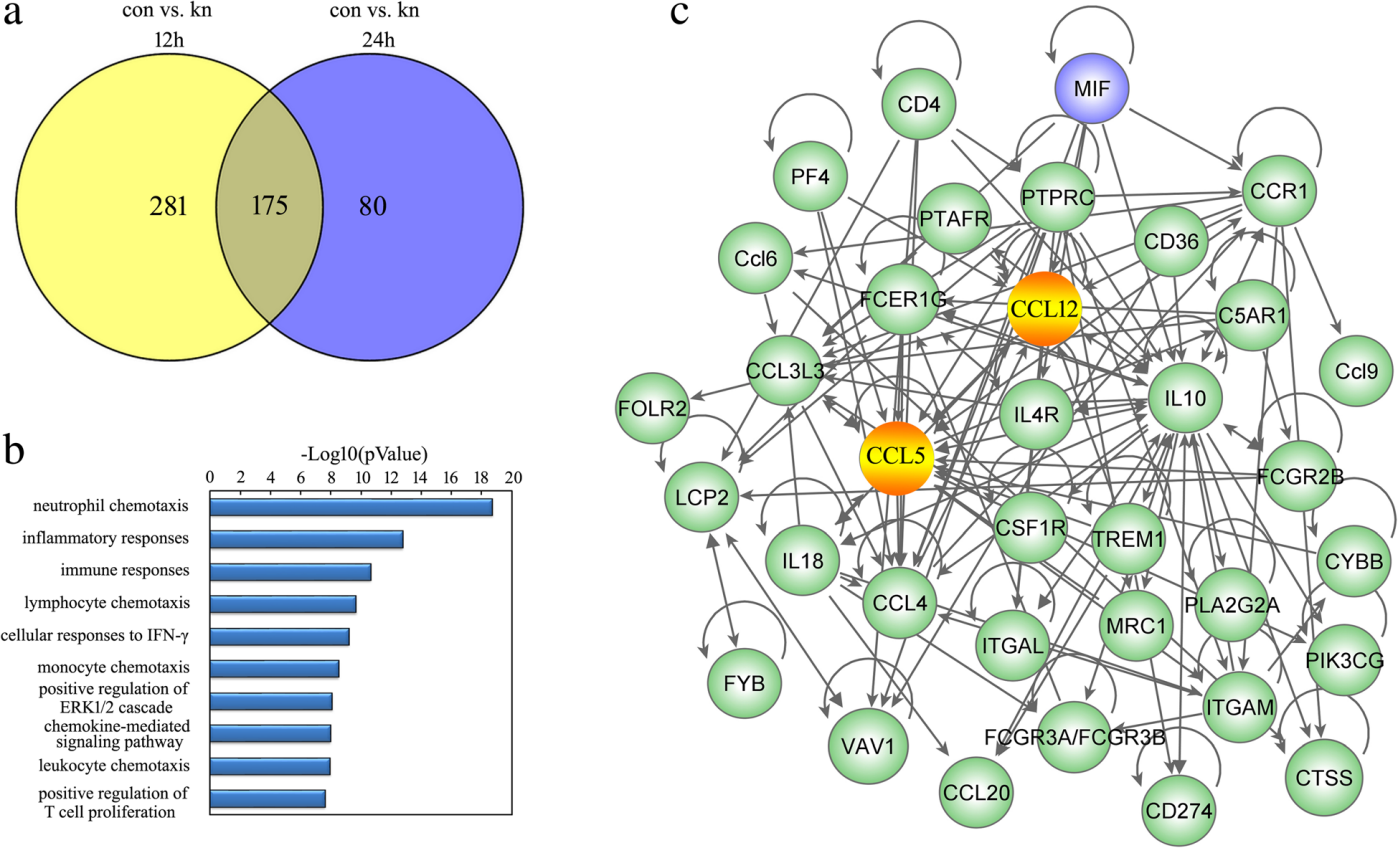
Functional annotations of DEGs and the inferred gene network in the astrocytes. **a** Integration of DEGs following knockdown of CD74 receptor for 48 h and then treated with 2 μg/ml recombinant rat MIF at 12 h and 24 h, respectively. **b** Most significantly enriched groups for the integrated DEGs relating to pathways. **c** A reconstructed gene network was created using the Ingenuity Pathway Analysis (IPA) Software on the basis of integrated DEGs

To understand whether CCL5 expression is under regulation of MIF/CD74 axis, we performed transcriptome analysis on primary astrocytes interfered by effective CD74 siRNA or scramble for 48 h [18], followed by stimulation with 2.0 μg/ml recombinant MIF protein for 12 and 24 h, respectively. A total of 456 and 255 differentially expressed genes (DEGs; siRNA versus scramble) were identified at the two time points, with defined criteria of *P* < 0.05 and a greater than twofold or less than twofold change (Fig. 3a). We further integrated the DEGs and characterized 175 functional genes (Fig. 3a). KEGG pathway enrichment analysis of these integrated DEGs revealed that chemotaxis signaling, inflammatory responses, and related ERK1/2 cascades were included in the significantly enriched functional pathway (Fig. 3b). Such pathways account for 48 of all 175 DEGs.

To shed light on the mechanism of molecular changes following blockade of CD74, the Ingenuity Pathway Analysis (IPA) was performed for the DEGs integrated at 12 and 24 h. The weight value of each gene in the network was calculated, representing the effect of MIF on the downstream targets. A reconstructed gene network showed that CCL5 and CCL12 with the highest weight value were highlighted as the important chemokines regulated by MIF/CD74 axis (Fig. 3c). As CCL12 has only been described in mice, we thus focus our attention on regulation of CCL5 [30].

**Fig. 4:**
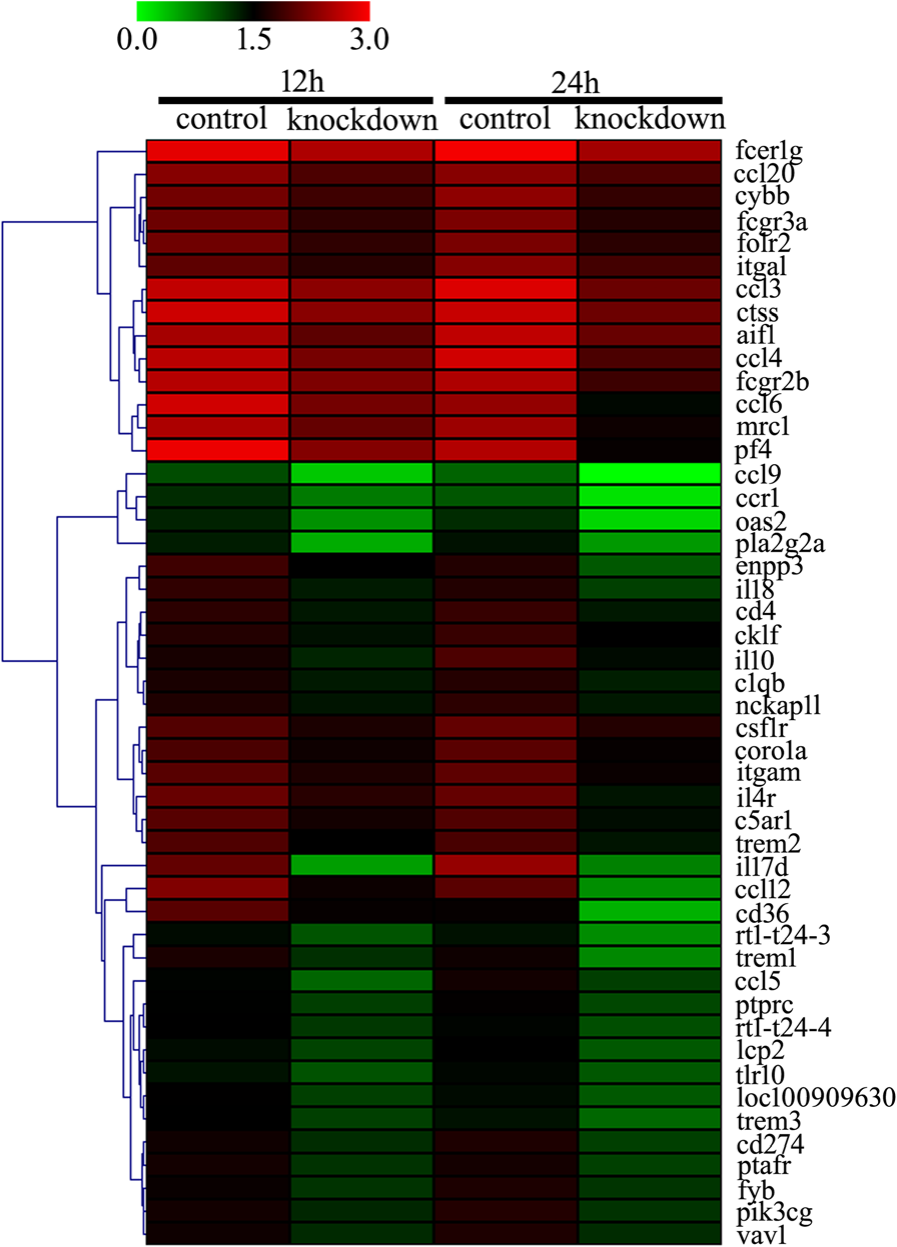
Heatmap and cluster dendrogram of integrated DEGs following knockdown of CD74 receptor for 48 h and stimulated astrocytes with 2 μg/ml recombinant rat MIF for 12 h and 24 h, respectively. The color scale shown at the top illustrates the relative expression level of the indicated mRNA across all samples: red denotes expression > 0 and green denotes expression < 0

All the genes displayed dynamic alteration following CD74 siRNA knockdown, as shown by the heatmap and cluster dendrogram (Fig. 4).

### MIF inhibitor attenuated CCL5 expression of astrocytes in vitro

**Fig. 5:**
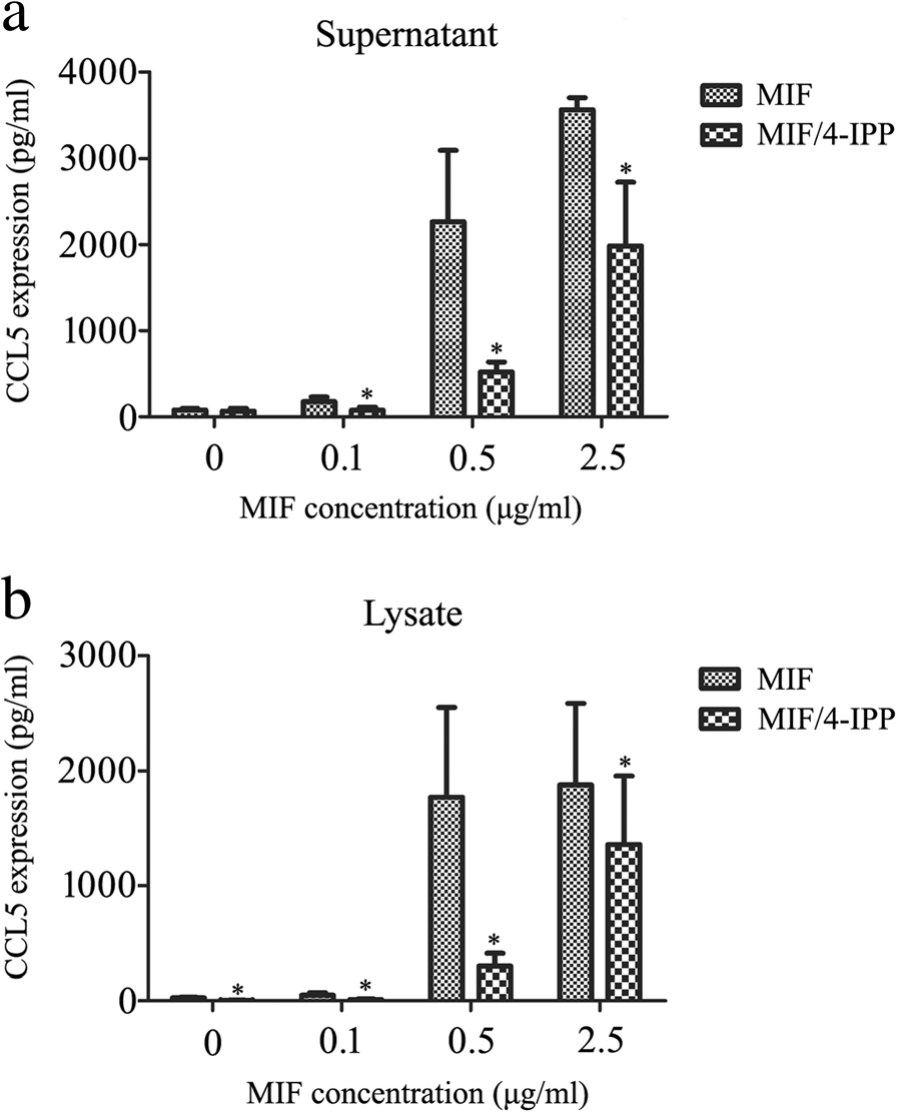
Examination of CCL5 production in astrocytes following stimuli with gradient recombinant MIF in the presence of 4-IPP. Cell supernatants (**a**) and lysates (**b**) were tested by ELISA for the chemokine CCL5, following astrocyte treatment with 0–2.5 μg/ml recombinant MIF for 24 h in the presence of 100 μM 4-IPP. Experiments were performed in triplicates. Error bars represent the standard deviation (*P* < 0.05)

To substantiate inference drawn from transcriptome analysis, the primary astrocytes were stimulated with recombinant MIF protein at a concentration of 0–2.5 μg/ml for 24 h, with or without addition of 100 μM 4-IPP. ELISA results showed that MIF was able to facilitate CCL5 production of astrocytes in a concentration-dependent manner. Treatment of astrocytes with 0.5 μg/ml MIF led to remarkable synthesis and secretion of CCL5, while addition of 100 μM 4-IPP to the culture significantly reduced CCL5 protein levels in both supernatant and lysate (Fig. 5a, b). It was interesting to note that 4-IPP even decreased protein levels of CCL5 in lysate in the absence of MIF stimulation, probably involved in inhibition of endogenous CCL5 expression. These results indicate that MIF directly induces the CCL5 expression in astrocytes.

### MIF promoted CCL5 production of astrocytes through JNK signaling

**Fig. 6:**
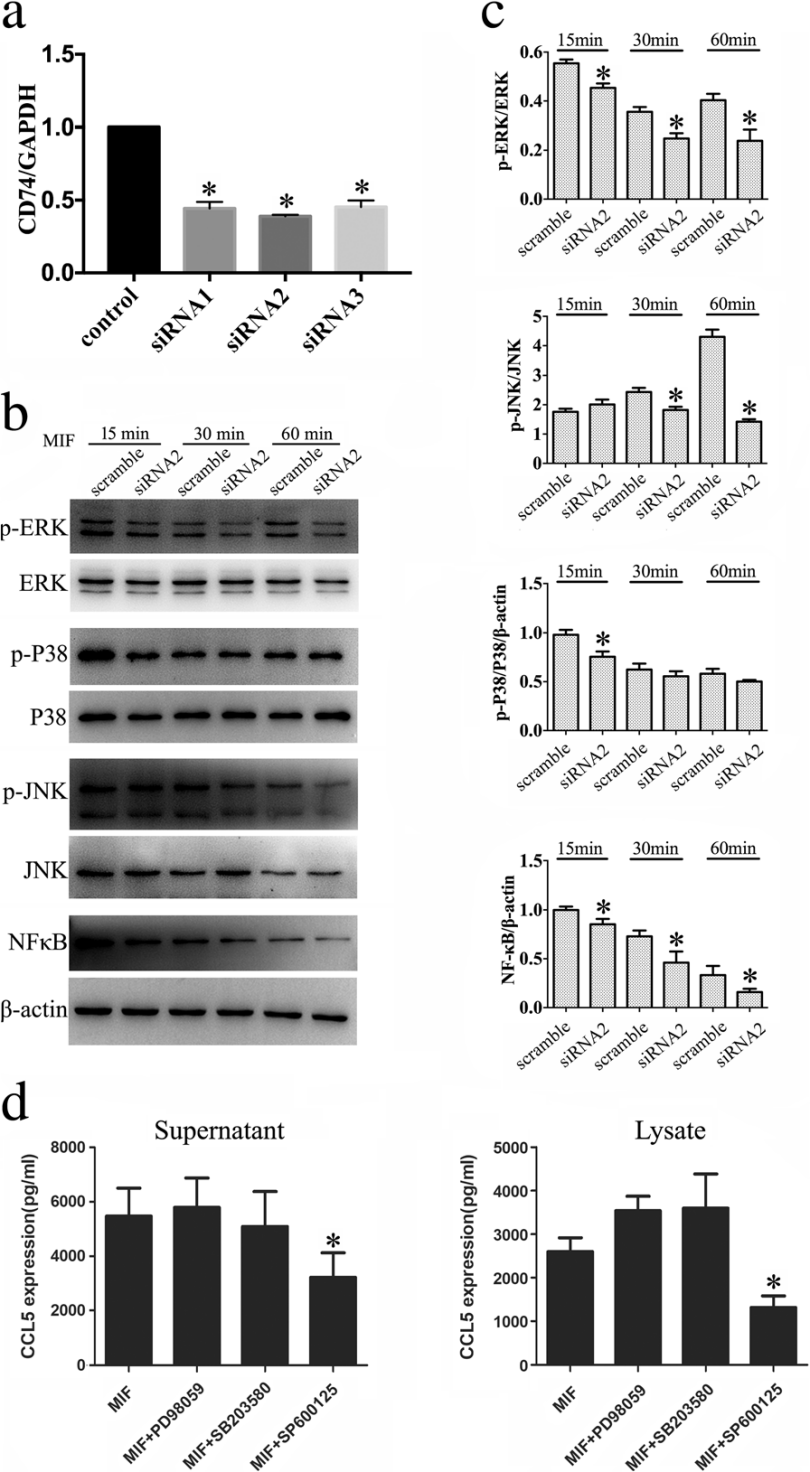
Determination of intracellular signaling associated with regulation of CCL5 expression. **a** Interference efficiency of three siRNA oligonucleotides for CD74 was measured by RT-PCR, and siRNA2 was used for the knockdown experiments. **b** Western blot analysis of pERK, p-P38, pJNK, and p65NFκB following astrocytes treated with siRNA oligonucleotides or scramble for 48 h and then with 2 μg/ml recombinant MIF for 15 min, 30 min, and 60 min, respectively. **c** Quantification data as shown in **a**. **d** Cell supernatants and lysates were tested by ELISA for the production of CCL5 following astrocyte treatment with inhibitor of ERK (PD98059), P38 (SB203580), or JNK (SP600125). Experiments were performed in triplicates. Error bars represent the standard deviation (*P* < 0.05)

Several virus, cytokines, or natural chemicals are able to induce CCL5 production in immune cells or glial cells through mitogen-activated protein kinase (MAPK) and NFκB signaling pathways [21, 31, 32]. To clarify the relevant pathway through which MIF facilitates CCL5 production of astrocytes, we interfered with the signal pathway through knockdown of CD74 receptor. The siRNA oligonucleotide (siRNA2) with nearly 60% knockdown efficiency of CD74 was chosen for the next experiments (Fig. 6a). CD74 receptor of astrocyte was knocked down by siRNA2 for 48 h, followed by the cell treatment with 0.5 μg/ml recombinant MIF protein for 15 min, 30 min, and 60 min, respectively. Determination of ERK, P38, and c-Jun N-terminal kinase (JNK) phosphorylation demonstrated that the phosphorylated level of these kinases and NFkB activity were significantly reduced in astrocytes (Fig. 6b, c). However, CCL5 production in the cells stimulated with recombinant MIF for 24 h was exclusively attenuated by treatment of JNK (SP600125), rather than by ERK (PD98059) or P38 (SB203580) inhibitor (Fig. 6d). These data indicate that MIF induces CCL5 production of astrocytes through JNK signaling.

### CCL5 primarily potentiated migration of IL-13-treated macrophages

**Fig. 7:**
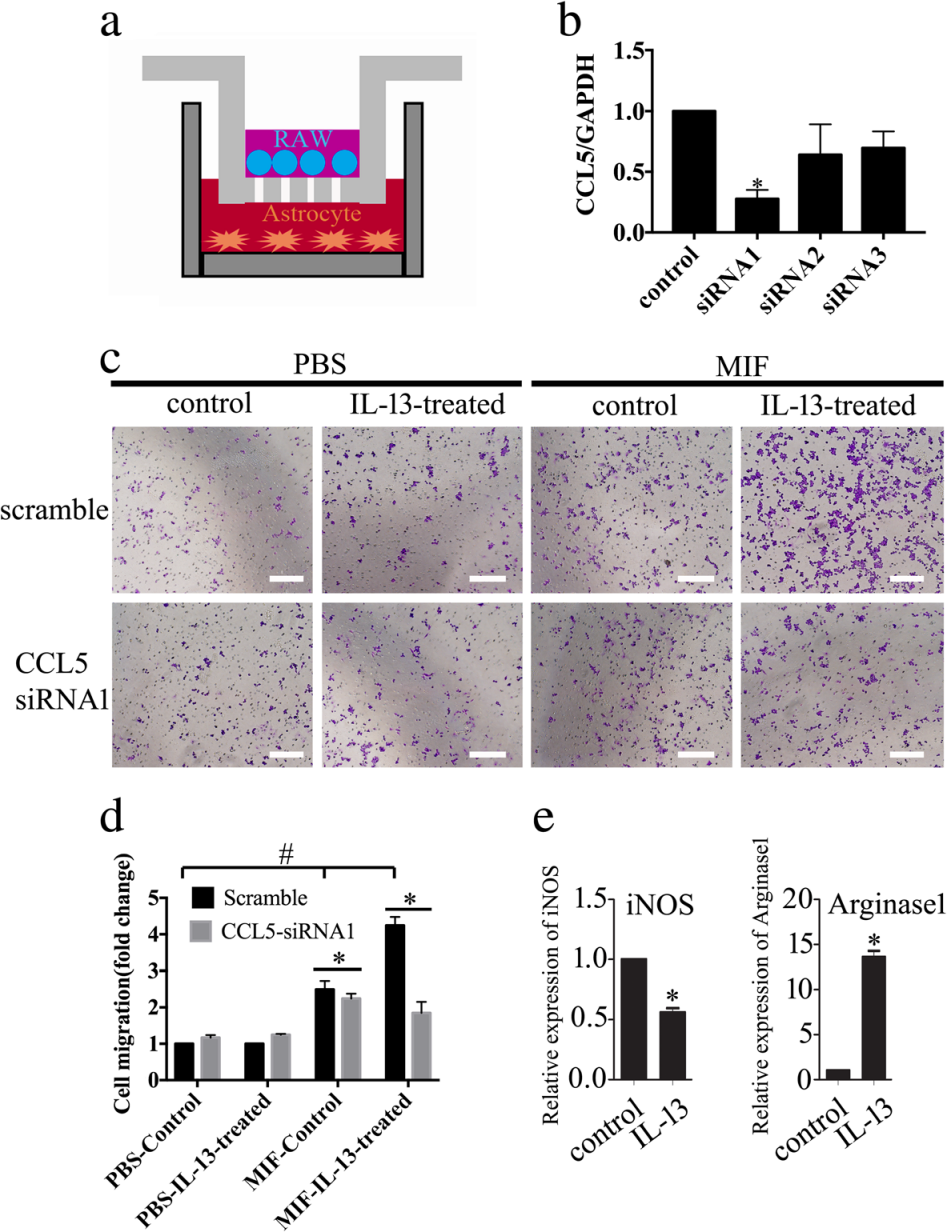
Effects of CCL5 on macrophage migration in vitro. **a** Transwell assay using cell co-culture model. **b** Interference efficiency of three siRNA oligonucleotides for CCL5 was measured by RT-PCR, and siRNA1 was used for the knockdown experiments. **c** Migration assay of macrophages co-cultured with astrocytes with or without knockdown of CCL5. Astrocytes were stimulated with 0.5 μg/ml recombinant MIF following CCL5 siRNA interference for 24 h prior to macrophage migration for 48 h. For a transition of macrophages from M1- to M2-phenotype, the cells were incubated in 20 ng/ml recombinant rat IL-13 protein for 2 days. **d** Quantification data as shown in **c**. **e** The expression of M1 and M2 macrophage markers, *iNOS* and *Arginase1*, was determined by RT-PCR. Quantities were normalized to endogenous β-actin. Error bars represent the standard deviation. *P < 0.05, ^#^*P* < 0.05. Scale bars, 100 μm

Chemokines are known to be essential for macrophage migration towards the site of injury [33]. To elucidate chemotactic function of astrocyte-released CCL5, we performed transwell assay to observe migration of macrophage RAW 264.7 cells in the presence of astrocytes with or without siRNA knockdown of CCL5 expression (Fig. 7a). Of three siRNA oligonucleotides, siRNA1 with a 72% CCL5 knockdown efficiency was selected (Fig. 7b). Astrocytes were stimulated with 0.5 μg/ml recombinant MIF following CCL5 siRNA1 interference for 24 h prior to macrophage migration for 48 h. Addition of recombinant MIF promoted a moderate migration of the unmodified macrophages, and such effect could be slightly attenuated by CCL5 siRNA1 interference, suggesting other factors in action (Fig. 7c, d). However, when the macrophages were incubated in 20 ng/ml recombinant rat IL-13 protein for 2 days, a transition from M1- to M2-phenotype identified by iNOS and Arginase1 expression [34], MIF was found to significantly facilitate the migration of these IL-13-modified cells (Fig. 7c–e). CCL5 knockdown of astrocytes remarkably reduced migratory cell numbers (Fig. 7c, d). The data indicate that CCL5 produced by astrocytes is primarily involved in promoting migration of M2 macrophages.

**Fig. 8:**
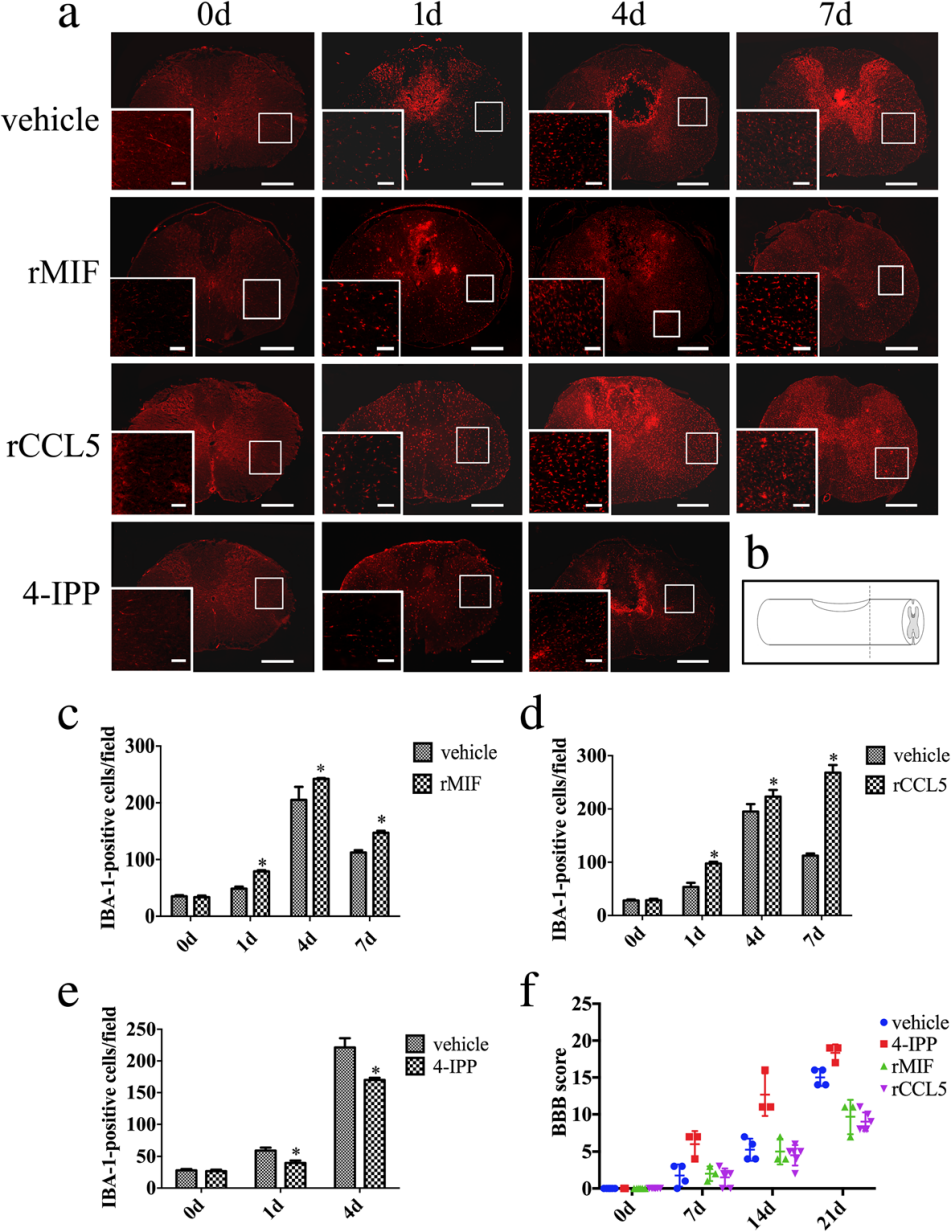
Immunostaining of microglia after rat spinal cord injury. **a** Immunostaining of IBA-1-positive cells following spinal cord contusion at 0d, 1d, 4d, and 7d, following injection of 8 μl of 100 μg/ml recombinant MIF, 4 μl of 100 μg/ml recombinant CCL5, or 8 μl of 100 mM 4-IPP, respectively. The rectangle indicates region magnified. **b** Illustration of section sites for immunostaining in the contused cord. **c**–**e** Statistic analysis of IBA-1-positive cells in **a** from 15 fields each. **f** BBB scores for hindlimb motor function in each group at different time points. Six rats/group were evaluated at 0d after injury, and four rats in the vehicle-treated group and three rats in the 4-IPP- or rMIF-treated group were evaluated from 7 days onwards due to sacrifice for immunostainings following significant difference (*n* = 3–6). Error bars represent the standard deviation (*P* < 0.05). Scale bars, 500 μm in section, and 100 μm in magnification

To observe the effects of MIF-induced CCL5 on migration of microglia in vivo, we detected the temporal distribution of microglia following spinal cord injury. Immunostaining with IBA-1 antibody displayed that SCI resulted in significant accumulation of microglia in the injured cord, with a peak at 4 days (Fig. 8a, c). Injection of 8 μl of 100 μg/ml recombinant MIF or 4 μl of 100 μg/ml recombinant CCL5 protein at the lesion site aggravated the amounts of IBA-1-positive cells. However, treatment with 8 μl of 100 mM 4-IPP resulted in a significant reduction of microglia migration (Fig. 8a–e).

To assess the influences of MIF or CCL5 on the functional recovery, BBB scores were measured during 3 weeks after the spinal cord injury. Injection of 8 μl of 100 μg/ml recombinant MIF or 4 μl of 100 μg/ml recombinant CCL5 significantly aggravated the hindlimb motor function compared with the vehicle. However, treatment of 4-IPP demonstrated a significant improvement (Fig. 8f). The results indicate that MIF-induced CCL5 release from astrocytes is sufficient to potentiate migration of microglia in the injured spinal cord, which is involved in the neuropathogenesis.

## Discussion

Emerging evidence has shown that MIF plays a role as a neuroimmuno-modulator in the CNS. Multiple cell types in CNS express this protein, such as microglia, fibroblasts, pituitary cells, endothelial cells, neurons, and neural stem cell progenitors [35]. Aberrant expression of MIF has shown to result in several pathological diseases of the CNS including Alzheimer’s disease, autism spectrum disorders, encephalomyelitis, and tumorigenesis [35–38]. In the acute phase of spinal cord injury, MIF contributes to neuropathological impairments through promoting neuronal death and inflammatory activation [14, 16]. It has been thought that microglia and other leukocytes are main resources of inflammatory cytokines in response to MIF stimuli [13, 39]. However, inflammatory signal pathway in astrocytes can also be activated by MIF through phosphorylation of ERK [18]. Here, we revealed a novel role of MIF in facilitating CCL5 production of astrocytes through activation of JNK, suggesting that MIF-induced releases of cytokines and chemokines are associated with multiple intracellular signals. Previous reports have shown that M1 and M2 macrophages populate the injury site with relatively equal distribution within the first week after SCI [40, 41]. MIF-induced CCL5 release from astrocytes is primarily implicated in the cell events of M2 macrophages.

MIF regulates distinct intracellular signaling in a cell type-dependent way. This factor has been shown to interact with CD74 receptor on macrophages, astrocytes, fibroblasts, tumor cells, or B lymphocytes, resulting in sustained activation of ERK1/2 kinase that brings in various effects [13, 18, 42]. Activation of MIF on JNK signaling has been identified in fibroblasts and T cell lines, in which phosphorylation of JNK is regulated by kinases PI3K and SRC [43]. In the CD11b^+^Gr-1^+^ myeloid cells, MIF is capable of potentiating chemotaxis, differentiation, and pro-angiogenesis through activation of p38/MAPK and PI3K/AKT signal pathways [44]. In the present study, we demonstrated that MIF/CD74 axis was able to regulate activities of ERK, P38, and JNK in astrocytes, suggesting a multifunctionality of MIF on astrocytes.

CCL5 induces the migration and recruitment of a wide variety of cells including T cells, dendritic cells, NK cells, eosinophils, basophils, mast cells, and endothelial progenitor cells [9, 45]. Interestingly, its effects on chemotaxis of macrophages are found to have tissue specificity. For examples, CCL5 is involved in recruitment and survival of macrophages in human adipose tissue [46] and in accumulation of macrophage in the kidney, liver, and peripheral nervous tissues [47–49]. In the CNS, however, the role of CCL5 in recruitment of inflammatory cells is controversial. In the hippocampi of mice, CCL5 has been shown to be not critical for accumulation of microglia [2]. We revealed that CCL5 primarily facilitates migration of IL-13-incubated macrophages, in consistency with that of IL-4-treated M2 macrophages [10]. The discrepancy may be attributed to the distinct phenotypes of microglia, either a greater recruitment of monocyte-derived macrophages in SCI or the alternative activation status stimulated by specific milieu [10, 50]. These results indicate that CCL5 alternatively facilitates accumulation of macrophages, depending on the cell subtype.

CCL5 is inducibly produced by invading pathogens or proinflammatory cytokines through activation of intracellular signals in a cell-specific manner [9, 31]. HIV-1 induces CCL5 primarily by transcriptional activation, while bacterial lipopolysaccharide (LPS) induces CCL5 in microglia via activation of TLR4 receptor [51]. Cytokine IL-1 is able to induce expression of CCL5 in astrocytes through phosphorylation of P38 or JNK, but not of ERK [31]. In the present study, we displayed that MIF induced CCL5 production in astrocytes through activation of JNK signaling. These results indicate that the regulatory mechanism of CCL5 expression is differential in different cell types.

## Conclusions

Protein levels of MIF were increased in response to SCI, which interacted with CD74 receptor of astrocytes to promote CCL5 production through activation of JNK. CCL5 in turn facilitated migration of macrophages to the lesion site, which affected repair of injured spinal cord.

## Additional file

Additional file 1: **Figure S1.** Determination of CCL5 colocalization with NeuN-, IBA-1-, or Olig2-positive cells following spinal cord contusion at 4 days. (PDF 397 kb)

## Abbreviations

ANOVA: Analysis of variance
MIF: Macrophage migration inhibitory factor
CCL5: Chemokine (C-C motif) ligand 5
PBS: Phosphate-buffered saline
MS: Multiple sclerosis
SCI: Spinal cord injury
GFAP: Glial fibrillary acid protein
ELISA: Enzyme-linked immunosorbent assay
KEGG: Kyoto Encyclopedia of Genes and Genomes
IPA: Ingenuity Pathway Analysis
DEGs: Differentially expressed genes
