# Supplementary material for: Macrophage migration inhibitory factor facilitates production of CCL5 in astrocytes following rat spinal cord injury
Source: J Neuroinflammation. 2018 Sep 4;15:253. doi: 10.1186/s12974-018-1297-z (PMC6122456; doi:10.1186/s12974-018-1297-z)
Supplement: Supplementary file 1 — Figure S1. Determination of CCL5 colocalization with NeuN-, IBA-1-, or Olig2-positive cells following spinal cord contusion at 4 days. (PDF 397 kb) [file 12974_2018_1297_MOESM1_ESM.pdf]

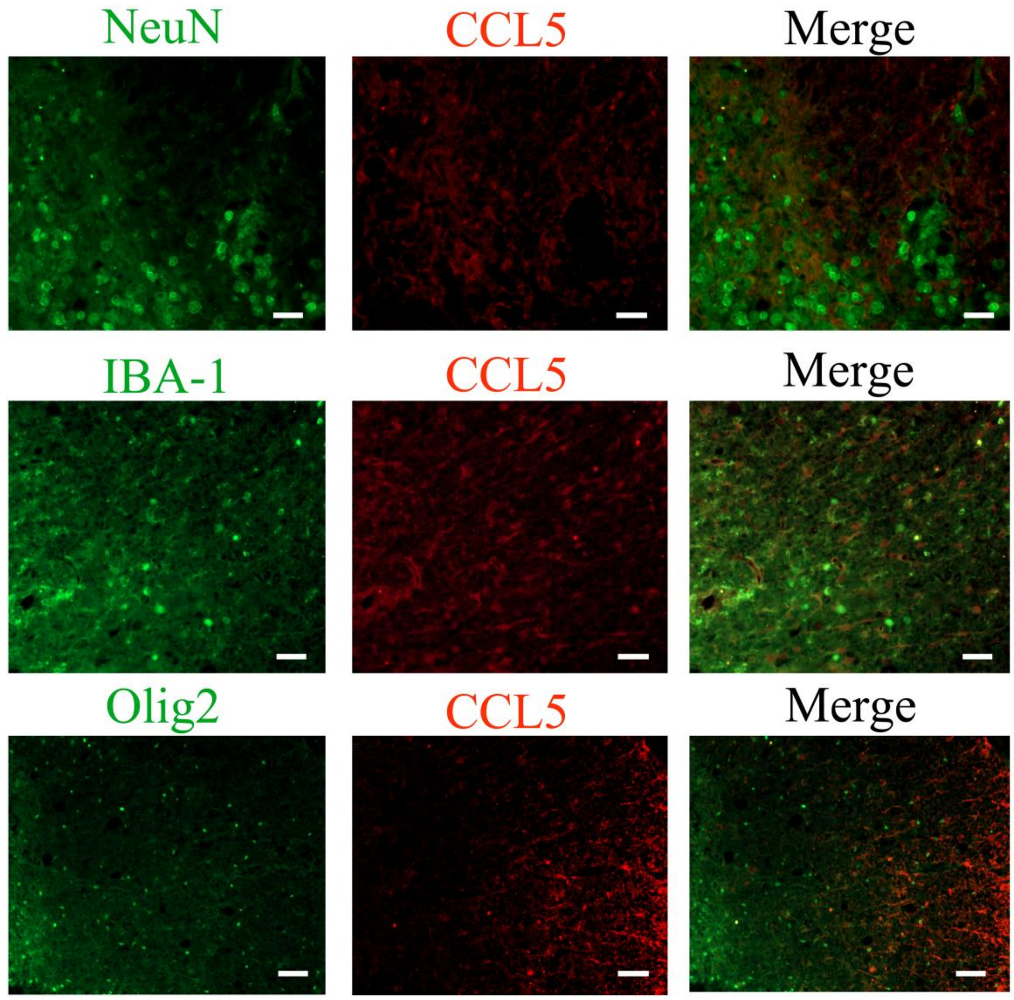

Fig.1 Determination of CCL5 colocalization with NeuN-, IBA-1- or Olig2-positive cells following spinal cord contusion at 4d. Scale bars, 50 μm.
